# Online professional development across institutions and borders

**DOI:** 10.1186/s41239-023-00399-1

**Published:** 2023-05-25

**Authors:** Bart Rienties, Blazenka Divjak, Michael Eichhorn, Francisco Iniesto, Gillian Saunders-Smits, Barbi Svetec, Alexander Tillmann, Mirza Zizak

**Affiliations:** 1 Institute of Educational Technology, The Open University, Milton Keynes, UK; 2 Faculty of Organization and Informatics, University of Zagreb, Zagreb, Croatia; 3 Aerospace Engineering Faculty, TU Delft, Delft, The Netherlands; 4 Studiumdigitale, Goethe University, Frankfurt, Germany; 5 Faculty of Medicine, University of Zagreb, Zagreb, Croatia

**Keywords:** Online professional development, Cross-institutional, Cultural, Mixed method, Innovative pedagogy, Higher education

## Abstract

Professional development (PD) is a key element for enhancing the quality of academic teaching. An increasing number of PD activities have moved to blended and online formats, especially since the COVID-19 pandemic. Due to the desire, potential, and need for collaboration among educators to learn from innovative and best practices, several institutions have started to pool their resources and expertise together and have started to implement cross-institutional and cross-national online professional development (OPD). The questions of what type of a (cross-)institutional OPD educators might prefer, and whether educators learn effectively from (and with) peers in such cross-cultural context have not been adequately explored empirically. In this case-study across three European countries, we explored the lived experiences of 86 educators as a result of a cross-institutional OPD. Using a mixed methods design approach our pre-post findings indicated that, on average, participants made substantial gains in knowledge. In addition, several cultural differences were evident in the expectations and lived experiences in ODP, as well as the intention to transfer what had been learned into one's own practice of action. This study indicates that while substantial economic and pedagogical affordances are provided with cross-institutional OPD, cultural differences in context might impact the extent to which educators implement lessons learned from OPD.

## Introduction

Providing appropriate professional development (PD) is essential to ensure that teachers have appropriate and up-to-date knowledge, competences, and skills to support their increasingly diverse students and design inclusive learning environments (Hsu & Lin, 2020; Mercader & Gairín, 2020). An increasing number of institutions in countries across the globe are providing online professional development (OPD) to complement and/or substitute face-to-face PD (Bragg et al., 2021; Dille & Røkenes, 2021; Lantz-Andersson et al., 2018; Macià & García, 2016; Rienties et al., 2013), in particular since COVID-19 (Bragg et al., 2021; Dille & Røkenes, 2021).

While there is a substantial body of literature on PD and OPD, most of these studies are either nested within one institutional context (e.g., Bragg et al., 2021; Howard, 2021; Powell & Bodur, 2019) or outside “institutional boundaries” in terms of providing informal PD via social media, MOOCs, and other communities of practice (e.g., Barrot & Acomular, 2022; Lantz-Andersson et al., 2018; Macià & García, 2016; Rehm et al., 2020). As institutions are recovering from the pandemic shock and the unprecedented move to online learning for their students and educators, several institutions are starting to structurally support cross-institutional programmes focussed on OPD and sharing expertise and insights beyond their own institutional boundaries (Bragg et al., 2021).

There are obvious potential benefits when designing and implementing OPD to pool resources and expertise together across institutions (Rienties et al., 2013), school districts (Kim et al., 2017), across a national level (Erixon, 2016), or perhaps even between institutions across geographical borders (Howard, 2021). Beyond economies of scale and efficiency arguments (i.e., costs savings) there are substantial opportunities for educators to learn from and network with colleagues from other institutions and countries (Rehm et al., 2020; Rets et al., 2020), which provide educators opportunities for reflection, agency, and meeting like-minded people (Barrot & Acomular, 2022; Howard, 2021; Lantz-Andersson et al., 2018).

At the same time, there is a wealth of literature highlighting that OPD needs to take into consideration individual needs and perspectives, and provide appropriate scaffolding (Dille & Røkenes, 2021; Howard, 2021). For example, Howard (2021, p. 2) indicated that “educators are more likely to disengage from courses that recycle content or do not account for differences in participants' needs and proficiencies, resulting in teachers exerting self-determination over the relevance of learning content, regardless of institutional mandates”.

This might be particularly relevant if educators are from culturally and geographically diverse contexts (Dennen & Bong, 2018; Jayatilleke et al., 2017; Zhang et al., 2016). In an adjacent field of cross-cultural psychology (Berry, 1992; Hofstede et al., 2010; Van de Vijver & Poortinga, 2002; Ward & Kennedy, 1994) there is a wealth of literature on how cross-cultural differences might support but also hamper communication, shared understanding, and collaboration between people from different cultures. However, while recently several systematic literature reviews have appeared on OPD (e.g., Bragg et al., 2021; Dille & Røkenes, 2021; Lantz-Andersson et al., 2018; Macià & García, 2016), perhaps surprisingly none of them specifically mention or incorporate culture and/or cross-cultural interaction between educators in OPD.

Therefore, in this study we specifically look at one cross-institutional OPD programme that was developed by four European universities in Croatia, Germany, the Netherlands, and the United Kingdom. In particular, we are interested to explore whether (or not) educators found these cross-institutional OPD programmes valuable, and whether (or not) there were differences in the lived experiences across educators from these four countries.

## Online professional development across institutional and national borders

### Benefits and challenges of OPD

There are several studies (e.g., Elliott, 2017; Yurkofsky et al., 2019) indicating that one of the main benefits of OPD for educators is that it can provide flexibility and (potentially) personalised choices. In OPD educators can consciously select respective activities of OPD they want to participate in, find meaningful, and perhaps most importantly, when and how they will engage. Furthermore, from an inclusive design perspective another powerful affordance of OPD is the opportunity for educators to engage with OPD activities even if they have accessibility needs, caring responsibilities, financial constraints, geographical constraints, etc. (Bragg et al., 2021; Elliott, 2017). In addition, OPD has the opportunity to provide personalised support and flexibility based upon the needs of educators (Azzolini et al., 2022). Finally, from an economic efficiency point of view providing OPD, in particular in a cross-institutional/national manner, might be more cost-effective than “traditional” face-to-face professional development (f-2-fPD) or single-institution PD provision (e.g., Azzolini et al., 2022; Bragg et al., 2021; Powell & Bodur, 2019).

Nonetheless, there could be several challenges when providing OPD in comparison to f-2-fPD or single-institution PD (Bragg et al., 2021; Powell & Bodur, 2019). For example, there are concerns that OPD may lead to relatively lower completion rates relative to f-2-fPD (Azzolini et al., 2022; Ma et al., 2020). It is well documented that online provisions, when not appropriately designed, might have lower retention rates due to a higher need of self-regulation and persistence (Bragg et al., 2021; Divjak et al., 2022b; Mercader & Gairín, 2020). Other scholars have debated whether educators are able to effectively transfer their OPD learning back into their own teaching and learning context (Bragg et al., 2021). For example, Powell and Bodur (2019, p. 20) argued that “access to [OPD] does not ensure quality experiences or outcomes and may create a false sense of effectiveness if technology is used merely as a delivery tool void of effective design or implementation principles”. Indeed Bragg et al. (2021, p. 2) indicated “OPD for teachers offers the potential for developing teacher knowledge by connecting them to a global community of peers with common professional learning goals who share resources and knowledge.”

Furthermore, in particular in cross-national OPD there might be a mismatch in terms of what educators from one country or context might need relative to another, or even what the preferred design of an OPD might be. For example, as indicated by Martin et al. (2019) who explored the lived experiences of OPD amongst 205 US instructors and 61 instructors from Germany and noted subtle differences in terms of expected pedagogical and technical support, which may be related to the national culture and state of curricula in respective countries.

In addition, regional and national educational policies might require specific educational goals of OPD programmes (e.g., levelling up in the UK, e-learning in education based upon e-school and e-university in Croatia) that may not be relevant for educators in other countries. In addition, there might be regulatory restrictions due to local or national legal requirements on academic teaching qualifications which may also limit participation in cross-institutional OPD activities. For instance, in the UK there is an expectation that academic staff work towards obtaining at a post-graduate certificate in academic practice (PGCAP) (Reimann & Allin, 2018). Although UK universities have agreed to mutually recognise each other's PGCAP, there is no formal recognition framework in place of other academic teaching PD offerings. As a result, each institution has its own process, rules and regulations on how to deal with non-recognised PD in academic teaching (Brouwer et al., 2022; Reimann & Allin, 2018).

### OPD and cross-cultural interaction

There is a relative paucity of research on effective cross-border OPD. For example, in a cross-cultural OPD context of 82 Canadian and Hong Kong pre-service teachers Zhang et al. (2016) found that some educators were able to deepen their situated understanding of multicultural education when working together in shared online spaces. At the same time, relatively few cross-cultural network links were established and maintained in these spaces (i.e., just around 1% of possible ties between the 82 pre-service teachers were developed).

In a cross-border OPD on online tutoring and mentoring lasting six weeks between 30 educators from Sri Lanka, Pakistan and Mauritius, Jayatilleke et al. (2017) found that while educators enjoyed the flexibility and opportunity to learn in OPD often there was limited (cross-border) peer engagement. In a four week OPD on social media in education, Dennen and Bong (2018) found strong national differences amongst 96 educators in terms of the expected course design and computer-mediated communication. For example, while “Western” participants felt comfortable with the interactive design, Chinese participants indicated a preference for a more lecture-oriented approach of the OPD, and were more reticent to share their lived experiences with non-Chinese peers. In a study of 167 HE educators from Croatia, Finland, Portugal, Spain, who participated in an online MOOC in autumn 2021 by Svetec et al. (2022) showed that substantial and significant differences across countries before and during the pandemic in terms of use of digital technology, teaching and learning strategies, and assessment practices.

In a recent RCT study of 3777 secondary school teachers from 511 schools in nine European countries and Turkey Azzolini et al. (2022) provided multiple personalised support offers (e.g., 20 min 1:1 session, reminder encouragement and advice) to three experimental groups relative to the control condition. The study found that personalised support offered improved probability of course completion of OPD with 10% for European professional teachers, but not for other groups (e.g., student teachers, those in Turkey). Whether or not cross-border interactions played a role in these differences was not explored.

### Research questions (RQ)

As indicated by the above literature, there is some emerging literature on how OPD can be used to connect educators across borders. However, most of the emerging literature is relatively small-scale and explorative in nature. To the best of our knowledge no study exists that specifically has looked at whether (or not) educators in a cross-institutional OPD grasp this potential to learn from peers in a cross-cultural context. Furthermore, in line with the above literature it is essential to determine whether all educators benefit from such cross-institutional OPD and collaboration opportunities with peers, or whether specific (sub-groups of) educators might be (dis)advantaged.

Therefore, our three research questions are:

1. To what extent do educators’ knowledge related to the aims of the Online Professional Development (OPD: i.e., use of innovative pedagogies of flipped classrooms/online work-based learning) improve as a result of a cross-institutional OPD?
2. To what extent do educators’ cultural backgrounds (i.e., their national background) influence their expectations and lived experiences in OPD?
3. To what extent do all educators benefit from OPD and social interactions with peers from different institutions, or are there specific sub-groups that might benefit more from OPD?

## Methods

### Setting and participants

This explorative mixed methods study aimed to analyse data collected from 86 Croatian, German, and UK educators enrolled into a short online course developed by four universities as part of an EU-funded Project RAPIDE (Relevant assessment and pedagogies for inclusive digital education). In its first e-course Module 1: Let’s innovate teaching! 141 participants registered while in the second e-course Module 2: Let’s innovate assessment 106 participants registered. Of those 114 engaged at least with the first quiz during a three-week period in June/July 2022 for e-course Module 1, and 70 engaged at least with the first quiz during a two-week period in September 2022 for e-course Module 2. The first of two e-courses, delivered in Moodle, specifically focussed on the innovative pedagogies of flipped classrooms (FC) and online work-based learning (WBL), while the second focussed on innovative assessment within these educational contexts.

**Fig. 1:**
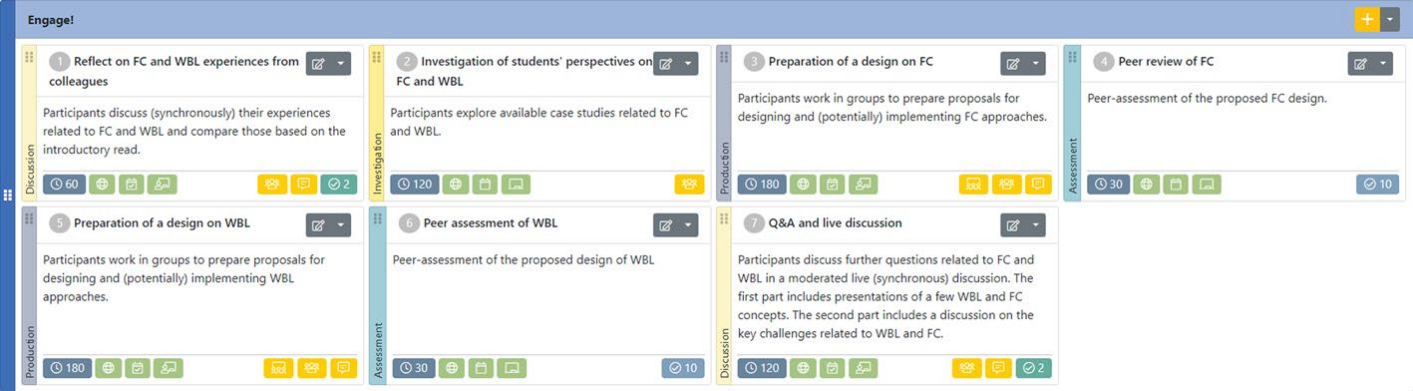
Overview of learning design of Engage! Phase in e-course Module 1

Educators worked fully online in three distinct phases. In the Prepare! phase primarily some basic literature on FC (Divjak et al., 2022b), WBL (Schuster & Glavas, 2017), and innovative assessment (Divjak et al., 2023) were provided, followed by two online quizzes and a subsequent live synchronous session in Zoom. In the Engage! phase participants reflected on their peers’ experiences of FC and WBL using case studies from the four institutions as well as their own experiences. As illustrated in Fig. 1, the design was flipped as participants were expected to come up with their own design creations.

Working together in smaller self-selected groups participants developed a (fictive) joined learning design in an innovative interactive tool called Balanced Design Planning (BDP: Divjak et al., 2022a; Rienties et al., 2023)). The expectation was that the learning design should at least include some element of FC or WBL. Again at the end of the week there was a synchronous Zoom session to discuss these initial designs. In total 32 designs were put forward by the 32 teams in the two e-courses, whereby 26 teams focussed on flipped classroom designs and six teams focussed on work-based learning.

Subsequently, groups updated their design based upon received feedback, and afterwards peer reviewed designs from at least two other groups. As a result, most groups received substantial peer feedback on their respective designs based upon their colleagues' perspectives and insights. In a last Zoom session, the final designs were briefly presented and a public and an expert award were given to the best designs.

Note, that when referring to educators in this study we specifically refer to participants of the two OPDs, while “teachers” of the OPD will be referred to as facilitators. All these activities took place online and while some educators might have met in their respective institutions incidentally most interactions between educators were solely based upon their online interactions.

### Instruments

#### Online survey on expectations (pre-measurement)

At the beginning of the e-course 108 educators completed a short twelve item survey about their respective role in their institution, their years of experience in teaching and learning, their expectations in terms of the e-course, and their respective experiences in FC, WBL, and other innovative approaches, and how many online courses they participated in the last two years. Of those 108, 86 educators were from Croatia, Germany, and the UK, who form our core focus in this article.

#### Online survey on lived experiences (post-measurement)

The lived experiences of the educators at the end of the e-course were measured via a second online survey consisting of twenty-six closed Likert Response items and five open items (e.g., What was the element of the e-course you liked most? What would you suggest we change or improve for the next implementation? In what ways will you adapt or change your practice as a result of studying this e-course?). The closed items focussed on the lived experiences of the design of the e-course (11 items, α = 0.889, e.g., “The materials, articles, and case-studies provided were useful for my learning”, “I enjoyed working with colleagues in this course”), the technology-acceptance of the BDP tool in particular (4 items α = 0.770, e.g., “I find it easy to get the BDP tool to do what I want it to do”), and the (potential) impact of the e-course on educators’ practice (8 items α = 0.874, e.g., “The course has encouraged me to use innovative teaching methods”). These three constructs were designed on purpose by the project team of Project XYZ based upon the above literature and research on learning design (Divjak et al., 2022a; Rienties et al., 2023). In total 79 educators completed this survey, of which 68 Croatian, German, and UK educators were included.

### Procedure and data analysis

Participants provided informed consent at the start of the e-course for research purposes to collect data in Moodle. Both pre- and post-survey were completed in Moodle which collects data in anonymous format. We consciously made this decision to allow participants to share any perspectives or insights without being worried that this might go back to their respective institution. Thus the reported results are on an aggregate level rather than on an individual level. The data were checked for normality using Shapiro–Wilk test and confirmed that the main constructs followed normality. Separate factor analyses were conducted on the pre- and post-test data separately, and indicated a reasonable fit of three lived experiences of the design of the e-course, (potential) impact on practice, and technology-acceptance. The three authors analysed the subsequent transcribed qualitative data from the surveys to identify key concepts reflecting the meanings attributed to the data (e.g., Lichtman, 2013).

## Results

### RQ1: To what extent do educators’ knowledge of innovative pedagogies (i.e., flipped classrooms and online work-based learning) improve as a result of a cross-institutional Online Professional Development (OPD)?

In total 108 educators completed the pre-survey at the start of the OPD, of which 86 were from the three countries involved in project RAPIDE. Note that no significant differences were found between the two e-courses, therefore we have aggregated the results across the two e-courses. Of the 86 respondents in total 67 academics/lecturers/teachers were included, four learning designers, eleven teaching support/professional support/academic related professionals, and four who did not classify them according to these three roles. 55% of educators had less than 10 years of teaching experience, while 45% of educators had more than 10 years teaching experience. On average, educators had participated in 2.88 online courses (SD = 2.81) in the last two years. This might seem relatively high but due to the pandemic a lot of educators had ample opportunities to follow institutional PD as well as other OPD.

In terms of prior knowledge of innovative practice of FCs and WBL, 30% of educators indicated to have insufficient knowledge, 48% indicated to have acceptable knowledge, while 22% indicated to have very good knowledge. In terms of actual usage before the start of the e-course, 46% of teachers indicated to rarely or never use FCs and WBL, while 20% (very) frequently used FCs and 31% (very) frequently used WBL.

**Fig. 2:**
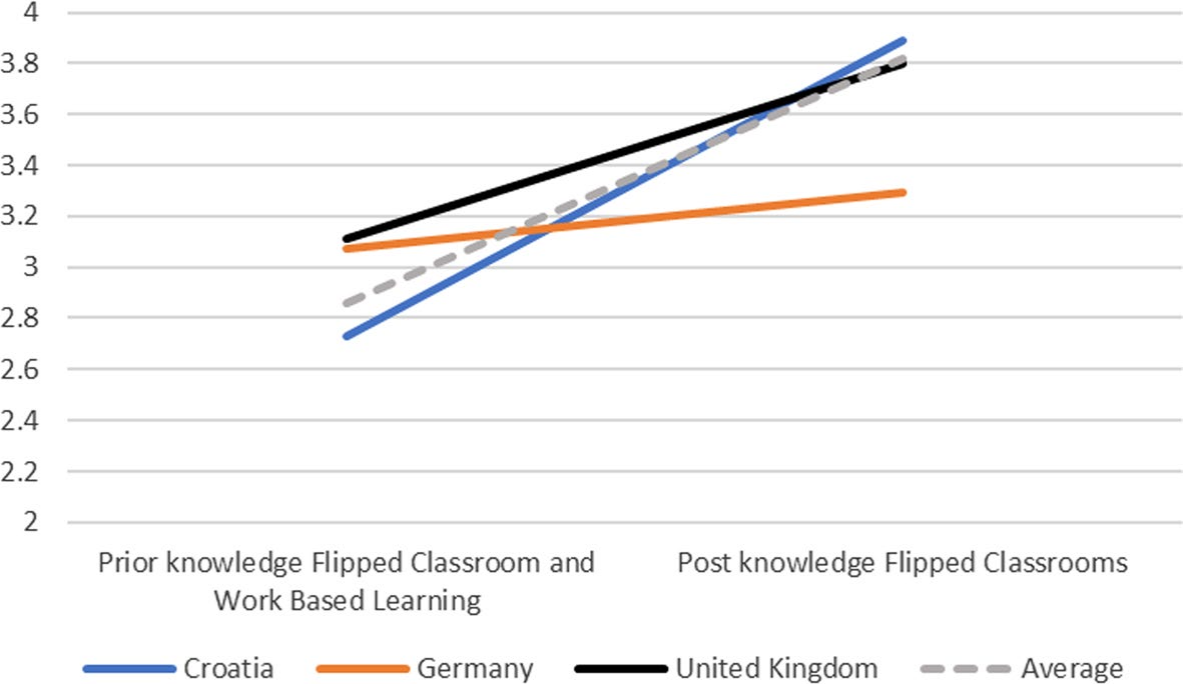
(Pre and post) Knowledge of flipped classrooms (1–5)

**Fig. 3:**
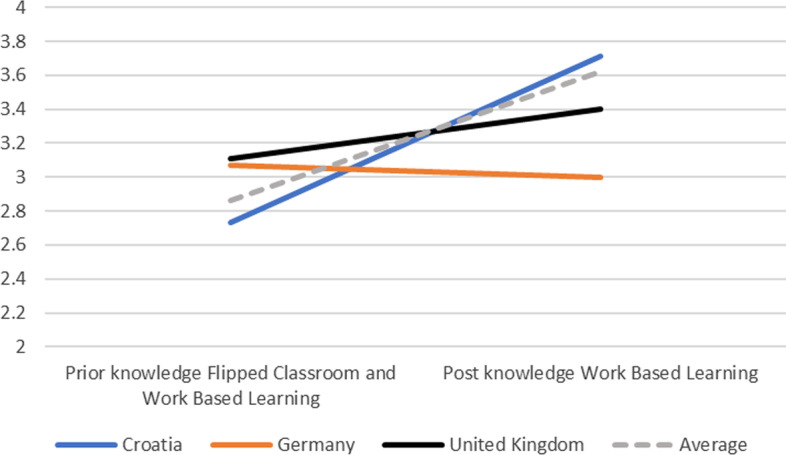
(Pre and post) Knowledge of work-based learning (1–5)

In terms of the evaluation at the end of the two e-courses, in total 79 educators completed the post-survey, of which 68 were from the three countries. 4% disagreed that they had knowledge of FCs, 30% were neutral, and 66% agreed or strongly agreed. Similarly, in terms of WBL 6% disagreed that they had knowledge of this innovative pedagogy, while 39% were neutral, and 54% agreed or strongly agreed. As indicated in Fig. 2, there was on average a substantial increase in knowledge in FCs, from 2.81 (SD = 0.90) to 3.82 (SD = 0.82), which according to a paired t-test was a significant increase (t = 10.151, p < 0.01, Cohen d = 0.80). As indicated in Fig. 3, similarly there was a substantial increase in knowledge in WBL, from 2.81 (SD = 0.90) to 3.63 (SD = 0.80), which according to a paired t-test was a significant increase (t = 8.415, p < 0.01, Cohen d = 0.79). In other words, there was a significant increase in knowledge in both FCs and WBL related to the e-course.

### RQ2: To what extent do educators’ cultural backgrounds influence their expectations and lived experiences in OPD

#### Prior expectations

As indicated in Table 1, there were several substantial cultural differences in prior expectations from educators when starting the OPD. On average, Croatian and UK educators had significantly higher expectations in terms of how the OPD would impact their practice relative to German educators. For example, Croatian educators had significantly higher expectations in terms of how the OPD will encourage them to use innovative teaching methods relative to their German or UK colleagues. Similarly, Croatian educators had higher expectations in understanding the impact of their teaching in the context of their institutional strategic goals. At the same time, Croatian teachers were less confident in terms of the prior knowledge of innovative pedagogies, as indicated in Figs. 2 and  3, and were less likely relative to their UK colleagues to use FC in their teaching practice.

**Table 1:** Prior expectations of participants in OPD e-courses

	Croatia		Germany		United Kingdom		F	η^2^
	M	SD	M	SD	M	SD		
Average prior expectations (0–1)	0.51	0.36	0.21	0.17	0.33	0.32	4.086*	0.090
The course will help me improve my teaching practice	0.93	0.25	0.88	0.35	0.78	0.43	1.821	0.042
The course will help me better evaluate my teaching competencies	0.63	0.49	0.38	0.52	0.50	0.51	1.271	0.030
The course will help me motivate students	0.52	0.50	0.13	0.35	0.28	0.46	3.436	0.076
The course will encourage me to use innovative teaching methods	0.45	0.50	0.00	0.00	0.28	0.46	3.668*	0.081
The course will support me in providing meaningful assessment in a digital environment	0.37	0.49	0.00	0.00	0.22	0.43	2.666	0.060
The course will help me understand learning data and its interpretation	0.32	0.47	0.00	0.00	0.17	0.38	2.382	0.054
The course will support me in understanding the impact of my teaching in the context of my institution's strategic goals	0.38	0.49	0.13	0.35	0.11	0.32	3.213*	0.072
How do you evaluate your pre-knowledge related to innovative pedagogical approaches in Flipped Classrooms and Work Based Learning? (1–5)	2.73	0.92	2.75	1.04	3.11	0.76	1.246	0.029
How often do you use flipped classroom in your teaching practice?	2.37	1.07	2.67	1.51	3.21	0.89	3.522*	0.085
How often do you use work-based learning in your teaching practice?	2.59	1.22	3.00	1.67	2.71	1.07	0.324	0.008

^*^p < 0.05, n Croatia = 60, Germany = 8, UK = 18

#### Lived experiences

As indicated in Table 2, the lived experiences of the design of the two e-courses were in general positive. 66 out of 68 (97%) respondents in the post-course survey were positive, in particular about the facilitators, the used e-course platform, and working with colleagues. In terms of the respective scales, the eleven items of the lived experiences of the design of the e-course (e.g., “The course has helped me improve my teaching practice”) indicated some subtle differences between Croatian, German, and UK educators. In general, Croatian teachers were more positive than their German and UK colleagues, although this was not significant based upon ANOVA.

**Table 2:** Lived experiences of participants at the end of the OPD e-courses

	Croatia		Germany		UK		F	η^2^
	M	SD	M	SD	M	SD		
(Average) Lived experiences of the design (1–5)	4.15	0.58	3.95	0.24	3.81	0.70	1.463	0.043
I learned a lot whilst studying this course	4.07	0.65	4.14	0.38	3.80	1.10	0.397	0.012
Overall I enjoyed participating in this course	4.07	0.75	4.00	0.00	4.00	0.71	0.044	0.001
The course has helped me improve my teaching practice	4.00	0.76	3.71	0.95	3.20	1.30	3.002	0.086
I enjoyed working with colleagues in this course	4.37	0.82	4.00	0.82	4.00	0.71	0.940	0.028
(Potential) Impact	3.96	0.58	3.54	0.46	3.37	0.95	3.684*	0.103
The course has encouraged me to use innovative teaching methods	4.18	0.69	3.71	0.95	3.20	1.64	3.655*	0.102
I have learnt how to evaluate my teaching competencies	3.91	0.72	3.57	0.79	3.20	1.48	2.348	0.068
I understand how to better motivate students	3.89	0.82	3.14	0.69	2.60	1.14	6.89**	0.177
Technology Acceptance of Balanced Design Planning	3.53	0.66	3.85	0.60	3.25	0.74	1.108	0.049

^*^p < 0.05, ** p < 0.01. n Croatia = 57, Germany = 6, UK = 5

However, the potential impact as measured by eight items (e.g., “The course has encouraged me to use innovative teaching methods”) was significantly higher for Croatian educators relative to German and UK educators, with a moderate to large effect size. In particular Croatian educators were more positive about how the e-course encouraged them to use innovative teaching methods and how to motivate their students relative to their German and UK educators. While there were some cultural differences in potential impact, all three groups of educators were similarly positive about working together with their colleagues in the e-course, and the BDP tool was considered useful. Obviously, the sample sizes for German and UK educators at the post-test were relatively small, which we will address also in our limitations.

### RQ 3: To what extent do all educators benefit from OPD and social interactions with peers from different institutions, or are there specific sub-groups that might benefit more from OPD?

In order to unpack some of the lived experiences between the three groups of educators beyond the quantitative data, we analysed the qualitative data from both the pre- and post-survey. We first analysed the data without taking into consideration the cultural background of participants. Afterwards, we reanalysed the data for each group separately and with three authors compared and contrasted notes. In order to sufficiently anonymise the data, we have randomly allocated a number to each educator and attached a starting letter to indicate their country (e.g., C5 is respondent 5 from Croatia, U9 is respondent 9 from the United Kingdom).

#### Lived experiences

In terms of the lived experiences, there was agreement that the overall structure of the e-course was innovative and appropriate. For example, C5 indicated “The very structure of the e-course, different types of activities (tests, workshop, work in groups, space for virtual work and group discussion, live sessions, BDP tool) and how they were carried out and how this contributed to the dynamics of work on the tasks and my motivation.”

This was echoed by C22 who, like several colleagues, enjoyed the flexibility of the course and the opportunities to engage with colleagues: “What I liked the most was the relaxed atmosphere and the flexibility in allocating time to create all the e-courses; planned activities. Additionally, great praise for the ability to communicate very quickly with the organisers and other participants involved in this e-course.”

At the same time, there were mixed perspectives in terms of the flexibility and assessment deadlines. As both e-courses were relatively short but intensive (i.e., 3 weeks) this was not necessarily appreciated by all. For example, several educators found that the flexibility of the e-course was at times difficult, in particular when these activities had to be combined with a busy work life. For example, C24 indicated “I don’t like fixed deadlines for assignment and teamwork, as well as live sessions, but it’s good that they are optional”. U9 indicated “I think it needs more time as all people are working during weekdays and many may be in different countries. This makes it a bit harder to coordinate everyone and results in some members not being able to work with the team, and incomplete work within the time given.”

At the same time several educators indicated that they enjoyed working with colleagues, such as C12: “Working with my colleagues on a new course, being creative, and learning how to use a tool to support our task”. This was supported by G10 who “liked working with the provided materials individually, but I also really enjoyed working in my group.”

At the same time, several educators found it difficult to work in groups and people from different institutions and cultures. For example, C6 indicated that “working in a group was disappointing for me because some people didn’t show up or participate. We had a lack of communication.” Similarly, C15 indicated that “the hardest thing for me was evaluating other works. They were not from my field of work, and besides, I don’t have enough experience and knowledge in working with a flipped classroom, especially with the results of analyses”.

#### Potential impact

In terms of how educators are going to implement some of the lessons learned from the two e-courses, several educators indicated that they gained confidence in implementing innovative pedagogies. For example, C4 indicated: “I have previous experience with the FC method (approx. 10 years ago) where I encountered difficulties because students would come to lectures unprepared despite the fact that they were evaluated by a test at the beginning of the class and they needed that prior knowledge in order to perform an exercise in class that also carried percentage of total grade. Encouraged by this course, I plan to introduce the flipped classroom method into the courses I teach, in certain units to begin with, to encourage my students to take a more active role in the teaching process. I believe that after the Covid pandemic and the experience with online teaching, this method would be more acceptable to students today and I hope that it will contribute to their motivation.”

Indeed 19 Croatian educators specifically mentioned that they would include and/or update the principles of FC in their next teaching practice. Seven Croatian educators also mentioned that they would include and/or update the principles of WBL in their next teaching practice. While some Croatian educators would start to implement FC in their next teaching practices, others like C32 used the e-course to reflect on their existing practice and fine-tune some of their course elements.

“To modify the existing subjects in which the mentioned pedagogical approaches are partially used so that they are even clearer (to each side, teachers and students) and to introduce some new activities that will undoubtedly improve the way of teaching, with the aim of the even better acquisition of both theoretical and practical knowledge, encouraging more students to work independently.” (C32).

The German educators were perhaps more cautious about the impact of the e-courses, with four colleagues like G32 indicated: “not sure yet”. Two German educators like G14 indicated that they would use some of the concepts used, such as “I will teach a FC course in autumn for the first time and this course helped me to understand what I need to do”.

The UK educators, like the German educators, were also more cautious in terms of how they would use the lessons learned from the e-courses. For example, U11 indicated “there is little space for innovation at an individual level where I work, but it does create food for thought”, while U3 indicated that they would “share my perspectives with my colleagues to inform training”. Only one out of five UK educators (i.e., U9) who completed the end survey indicated that they were “planning my courses with a more student-centred approach and how I can adapt the activities so we maximise learning opportunities when we meet in synchronous activities”.

#### BDP tool

In terms of the BDP tool that was used to develop a joined group learning design seven Croatian educators (e.g., C8) indicated that the BDP tool was the most useful element of the e-course, while similarly G14, U3 and U9 found the BDP one of the most useful elements of the e-course. For example, U9 indicated “The BDP tool was new to me and I enjoyed being able to have a more visual representation of my plan. It helps me make sure I am keeping the activities and goals balanced and I loved how easy it is to edit the project there.” Four Croatian educators (i.e., C4, C6, C20) specifically indicated that they would use the BDP tool in their next teaching practice, while none of the German or UK educators specifically mentioned that they would use the BDP tool.

In other words, while there seems to be general agreement that educators found the e-courses useful, innovative, and engaging. At the same time, as previously highlighted in the quantitative findings several Croatian educators seemed to be more positive about the lived experiences of the e-courses, and seemed more inclined to implement the lessons learned into their teaching practice relative to their German and UK colleagues.

## Discussion and conclusion

There is some emerging literature on how Online Professional Development (OPD) can be used to connect educators across institutional and national borders (Bragg et al., 2021; Jayatilleke et al., 2017; Lantz-Andersson et al., 2018; Zhang et al., 2016) to help them to share and learn about innovative practice. There are several economic and social arguments why providing cross-institutional OPD might not only be financially attractive (Azzolini et al., 2022; Bragg et al., 2021; Rienties et al., 2013) but also might encourage sharing innovative practice beyond the borders of institutions and nations (Dennen & Bong, 2018; Lantz-Andersson et al., 2018; Macià & García, 2016; Rienties et al., 2023). At the same time, working together in different cultural and national OPD contexts might not always be straightforward (Dennen & Bong, 2018; Jayatilleke et al., 2017; Martin et al., 2019), and local/national educational policies might impose country-specific requirements in terms of which educational goals need to be covered.

As indicated in this explorative mixed method study, educators' knowledge of innovative pedagogy in terms of flipped classrooms (FC), online Work Based Learning (WBL), and assessment improved significantly over time (RQ1). The 68 educators from three countries (i.e., Croatia, Germany, the UK) rated their knowledge significantly higher after attending the two e-courses in both FC and WBL than at the beginning of OPD. In other words, the jointly designed e-courses were effective in increasing knowledge and expertise in innovative pedagogy.

With regard to the expectations of the OPD and the educators' lived experiences (RQ2), some cultural differences became apparent in both quantitative (pre-post) data as well as qualitative data, in line with previous findings (Dennen & Bong, 2018; Jayatilleke et al., 2017; Zhang et al., 2016). Thus, educators from the three countries estimated their prior knowledge of innovative teaching formats differently, but in return also expected different degrees of impact of the OPD on their teaching practice. Croatian educators significantly improved their knowledge of innovative teaching formats relative to German and UK educators, and were also more inclined to implement them into their own practice. It can be observed that when prior knowledge tends to be rated lower, expectations are higher in terms of improved teaching practice and deeper understanding of the connections between teaching and HEI strategic goals.

With regard to RQ3, it can be noted that educators unanimously stated that they found the OPD and e-courses useful, innovative, and engaging. This is especially true for the course design, facilitation, and collaborative small group work consisting of educators from different countries. Regarding the workload as well as flexibility, e.g., in submissions and assessment deadlines, educators indicated some different perspectives. For example, the very intensive schedule of the e-courses was not equally appreciated by all, and it was sometimes difficult for educators to reconcile with their daily work routine.

It was also evident from the qualitative data that the Croatian educators in particular rated the learning experiences in the OPD more positively than the German and UK educators, and were also more likely to indicate that they would like to apply the competencies acquired in the course in their teaching practice. In other words, while there are definitively positive economic, social, and pedagogical reasons to consider to implement cross-institutional and perhaps cross-national OPD, our findings seem to suggest that not all groups of educators equally benefited from such approaches.

This is line with a wealth of cross-cultural literature, indicating that what might work for one culture may not necessarily also work for another (Berry, 1992; Hofstede, 1986; Ward & Kennedy, 1994; Zhang et al., 2016). For example, over 40 years of work on national cultures by Hofstede et al. (2010) has shown that national educational programmes have a strong influence on national culture, and how people from one country relative to another country, for example, cope with power distance, uncertainty avoidance, and long-term orientation. While we are mindful of stereotypes, Croatians tend to have a stronger power distance than Germans and British people, while the UK scores relatively high on individualism, and Germans have relatively high scores on long-term orientation. Furthermore, there was stronger support from senior management in Croatia for participants who joined the e-courses, while at the same time relatively more educators from competitive disciplines like medicine and IT joined the cross-national OPD. This might explain why Croatian educators were perhaps more inclined to indicate that they would rapidly implement these innovations in their practice. In contrast, perhaps the German educators might take perhaps a slightly more conservative approach, while in the UK academics have substantial freedom to make educational decisions in terms of learning design. Obviously without detailed follow-up qualitative interviews (which goes beyond this study) it would be difficult to assess the complex and multiple reasons why educators across the three countries made different decisions, but cultural factors seem to play a mediating role.

### Limitations and practical implications

Given the increased affordances and (financial) pressures to offer cross-institutional OPD, our findings indicate some substantial differences in lived experiences, indicating that a one-size fits all approach might not necessarily work. Obviously, our research has several limitations. First of all, while in comparison to other OPD research studies we were able to recruit a relatively large cross-cultural cohort of teachers in three countries, the imbalance of the sample size of the three cohorts, the relative small sample size of the German, UK, and Dutch educators, and the relatively low response rates of educators at the post-test might limit the generalisation of these findings. There could be a variety of reasons why engagement rates by these three groups of educators were not homogeneous, including differences in institutional support, disciplinary background, and individual motivation. As these OPD e-courses were offered for free within the participating institutions of RAPIDE and were not linked to formal recognition schemes, future research should explore whether these reported cultural differences remain pertinent when these OPD e-courses become embedded into formal OPD.

A second obvious limitation is that we only used self-reported quantitative and qualitative data, with inherent biases. Future research should explore with larger samples and actual measurements of implementation of (future) practices whether Croatian educators were indeed more inclined to implement these practices following the OPD, or whether cultural response styles (Van de Vijver & Poortinga, 2002), the way respondents from particular cultural backgrounds complete surveys, might explain part of these differences.

The key practical implications of this research are:

1. Cross-institutional OPD need to focus on practical experiences that can enrich and strengthen educators’ knowledge, awareness, and understanding of innovative teaching practice (without missing contextualising aspects).
2. Expectations of educators from varied backgrounds influence both expectations and impact of OPD. That is something to be considered when creating these types of cross-institutional OPD in international contexts.
3. Participating in e-courses with an international audience benefits from the cooperation in terms of sharing experiences, which may not only differ from the cultural background but from coming from different disciplines and using different procedures to prepare pedagogical content based on flipped classroom and work-based learning.
4. Researchers conducting cross-institutional OPD data analyses need to be mindful that cultural differences might substantially impact reported findings, which might require either sub-sample analysis or controlling for culture when modelling learning processes and performance.

## Data Availability

The datasets used and/or analysed during the current study will be made available in an online repository after acceptance of this manuscript. The study has undergone appropriate ethics protocol at University of Zagreb. Informed consent was sought from the participants. Authors consented the publication. Participants consented to publication as long as confidentiality is observed.
